# Mindfulness-based cognitive therapy in obsessive-compulsive disorder – A qualitative study on patients’ experiences

**DOI:** 10.1186/1471-244X-12-185

**Published:** 2012-10-31

**Authors:** Elisabeth Hertenstein, Nina Rose, Ulrich Voderholzer, Thomas Heidenreich, Christoph Nissen, Nicola Thiel, Nirmal Herbst, Anne Katrin Külz

**Affiliations:** 1 Department of Psychiatry and Psychotherapy, University Medical Center Freiburg, Hauptstraße 5, Freiburg, 79104, Germany; 2 Schön Klinik Roseneck, Prien, Germany; 3 Esslingen University of Applied Sciences, Esslingen, Germany

**Keywords:** Obsessive-compulsive disorder, Mindfulness, MBCT, Complementary treatment, Qualitative content analysis, Subjective experience

## Abstract

**Background:**

Cognitive behavioral therapy (CBT) with exposure and response prevention (ERP) is the first-line treatment for patients with obsessive-compulsive disorder (OCD). However, not all of them achieve remission on a longterm basis. Mindfulness-based cognitive therapy (MBCT) represents a new 8-week group therapy program whose effectiveness has been demonstrated in various mental disorders, but has not yet been applied to patients with OCD.

The present pilot study aimed to qualitatively assess the subjective experiences of patients with OCD who participated in MBCT.

**Method:**

Semi-structured interviews were conducted with 12 patients suffering from OCD directly after 8 sessions of a weekly MBCT group program. Data were analyzed using a qualitative content analysis.

**Results:**

Participants valued the treatment as helpful in dealing with their OCD and OCD-related problems. Two thirds of the patients reported a decline in OCD symptoms. Benefits included an increased ability to let unpleasant emotions surface and to live more consciously in the present. However, participants also discussed several problems.

**Conclusion:**

The data provide preliminary evidence that patients with OCD find aspects of the current MBCT protocol acceptable and beneficial. The authors suggest to further explore MBCT as a complementary treatment strategy for OCD.

## Background

“Mindfulness” can be described as a process of purposely bringing attention to moment-by-moment experience in a non-judgmental way [1]. Mindfulness-based cognitive therapy (MBCT) [2] is a manualized 8-week group clinical intervention program combining mindfulness training with elements of cognitive behavioral therapy (CBT), developed to reduce relapse in patients who are in remission from major depressive disorder.

Clinical trials of MBCT have found significant symptom reduction in patients with affective disorders and some anxiety disorders. Two recent reviews report medium effect sizes of MBCT compared to control conditions [3,4]. However, little is known about how MBCT works and whether its benefits stem from specific components of MBCT or unspecific effects of a group intervention [3].

One way of exploring the effects of different components of an intervention is drawing on the subjective experience of patients who undergo the treatment. This method seems especially suitable in the field of mindfulness research, as mindfulness training particularly focuses on patients’ subjective awareness and how they experience thoughts and feelings [5].

Several studies have already examined how patients with mood and anxiety disorders experience MBCT, using qualitative methodology. Finucane and Mercer [6] reported that a majority of thirteen patients with recurrent depression, who were interviewed three months after the course, experienced MBCT as both acceptable and beneficial. Allen et al. (2009) studied twenty patients in full or partial remission from major depressive disorder twelve months after MBCT. Four over-arching themes emerged from their data: control, acceptance, relationships and struggle. Bailie, Kuyken and Sonnenberg [7] focused on MBCT’s impact on the parenting behavior of 16 participants with recurrent depression. Their results showed that all but one participant continued mindfulness practice over the course of one year. The authors observed that mindfulness practice was accompanied by changes in participants’ interaction patterns with their children, such as an improved management of negative emotions in challenging situations, an increased ability to adopt their childrens’ perspective and an enhanced recognition of own needs. Another qualitative study by Chadwick et al. [8] indicated that patients with bipolar disorder might also benefit from MBCT. This mostly applied to a reduced impact of the current mood state and a higher acceptance of mood changes. Making use of interpretative phenomenological analysis, Williams et al. [9] observed a beneficial effect of MBCT in patients with hypochondriasis, both with regard to symptoms of health anxiety and broader functioning. Yet, to date, there are no publications on experiences of patients with OCD who receive MBCT.

OCD which is characterized by distressing intrusive thoughts and time-consuming compulsive rituals, is a common psychiatric disorder with a lifetime prevalence of 1 to 3% [10]. CBT including exposure and response prevention (ERP) is the first-line treatment for OCD (e.g. [11,12]). Augmenting treatment with selective serotonin reuptake inhibitors (SSRI) is recommended for patients without overt compulsions, those who do not respond to CBT alone or those with comorbid major depression [13]. However, in a recent investigation of an extensive therapy program including daily therapist-assisted exposure and response prevention exercises over a period of three weeks, only 55% of the patients could be classified as treatment responders when response was defined as a decrease of symptom severity by at least 35% [14]. In a systematic review of psychological and pharmacological treatment studies, it was found that symptoms persisted at a moderate level following adequate treatment [12].

Due to the anxiety-provoking nature of ERP, patients often perceive the therapy as demanding. In MBCT, in contrast to ERP, patients are encouraged to observe unpleasant experiences as they arise and label them as thoughts, feelings or bodily experiences, but unpleasant experiences are not provoked intentionally. In contrast to traditional cognitive therapy (CT) (see e.g. [15]) that aims to identify and restructure maladaptive thoughts, MBCT rather targets the attitude towards thoughts, not their content. That is, in MBCT, patients are taught to carefully observe every arising thought, label it as a thought, try not to judge it and refrain from acting on it compulsively.

Supplementing existing evidence-based treatments with mindfulness-based interventions has repeatedly been recommended for patients with OCD, for example by Didonna and Fairfax [16,17]. Preliminary results point towards the efficacy of mindfulness-based treatments for the reduction of OC symptoms: In a quasi-experimental study on students, a decline of subclinical OC symptoms was observed after a mindfulness based intervention [18]. A randomized controlled trial has demonstrated that acceptance and commitment therapy (ACT), an intervention which employs mindfulness as well as a number of other strategies, is superior to relaxation training for patients with OCD and might produce a similar reduction in symptoms as CBT [19]. However, as ACT is not solely based on mindfulness but includes numerous other therapeutic interventions such as the clarification of values and committed action, conclusions on the efficacy of mindfulness for patients with OCD cannot be drawn from these results. The authors of a recent review concluded that mindfulness holds promise as an intervention for the treatment of OCD, but the research in this area is still at a very early stage [20]. It remains to be further elucidated whether MBCT as an adjunctive therapy to CBT with ERP might be useful to address some of the challenges mentioned above.

The broad aim of our current research program is to investigate whether MBCT represents a feasible and effective, complementary treatment option for patients with OCD who are familiar with the principles of CBT including ERP. Here we report on a pilot study that applies qualitative methodology to provide further insight into the subjective experiences of OCD patients participating in eight group sessions of MBCT. Particularly, we investigated i) subjective changes of experience and behavior within an eight-week period, ii) helpful and problematic aspects of MBCT and iii) suggestions for the adaptation of the program to the specific needs of patients with OCD.

## Methods

### Ethical approval and informed consent

The study was approved by the ethics committee of the Albert Ludwig University Freiburg. Informed written consent was obtained from each subject prior to the onset of the study.

### Study participants

Sixteen subjects with OCD according to DSM-IV criteria who had completed a behavioral therapy with ERP within a two-year period prior to the study were included. Four subjects dropped out during the first three treatment sessions: one due to difficulties in scheduling appointments, another two because they re-initiated a behavioral therapy, and one in whom a traumatic brain injury was discovered. Twelve subjects completed the MBCT intervention and took part in semi-structured interviews within two weeks after the course. The analyzed sample (N = 12) consisted of three women and nine men with a mean age of 41.8 years (SD 12.4 years) who, on average, had suffered from OCD for 19 years. At baseline, their average score was 17.7 points (SD 8.1, range 7 to 28) on the Yale Brown Obsessive Compulsive Scale [21,22], a well established instrument measuring the severity of OCD. 17.7 points equal a moderate degree of OCD. The most commonly reported major symptoms were concerns with dirt and contaminants and washing/cleaning compulsions. Exclusion criteria were the current diagnosis of a severe depressive episode, substance abuse, borderline personality disorder, current or past diagnosis of a psychosis and severe brain injury, as well as age outside the range of 18 to 65 years and current psychotherapeutic treatment. Diagnoses were made by a trained psychologist according to DSM-IV criteria, using the Structured Clinical Interview for DSM-IV [23]. Six subjects were on medication, mainly selective serotonin re-uptake inhibitors. In all subjects, the medication status had been stable for at least ten months prior to inclusion. Three patients fulfilled criteria for a personality disorder, namely avoidant, histrionic and paranoid personality disorder.

### Intervention

The intervention program was adapted from “MBCT for depression: a new approach to preventing relapse” as described by Segal, Williams and Teasdale [2]. Cognitive elements of the manual were adapted for OCD with the objective of providing a preferably disorder-specific treatment. Mindfulness elements were adopted from the original manual. MBCT was delivered in eight two-hour-sessions, once a week. Each session included brief reporting on the previous week, a review of weekly “homework” (mindfulness practice outside the sessions), guided in-session mindfulness practice and exchange within the group as well as a preview of the “homework” for the following week. After each session, participants were given handouts with a summary of the most important session contents. They were also given two CDs with instructions for mindfulness exercises they could practice at home. Table 1 provides a summary of the major learning objectives of MBCT and corresponding contents of the course. OCD-specific interventions are highlighted in bold type.

**Table 1:** Major learning objectives of MBCT and course contents

**Objective**	**Course contents**
Becoming familiar with some characteristics of the mind	introducing “the autopilot” as a symbol for a state of mindlessness
	**introducing** “**the spectacles of OCD**” **as a symbol for selective information processing and cognitive distortion**
Exploring new ways of refocusing attention and challenging dysfunctional meta-cognitive beliefs	the “body scan”, a 45-minute mindfulness exercise teaching participants to attentively and non-judgmentally become aware of their body and body sensations
	yoga exercises
	dealing with arising thoughts and feelings during sitting meditation exercises
	the three-minute-breathing-space, a short exercise in which patients are invited to focus their attention on their breathing and present momentary experiences, shifting from a “doing-mode” to a “being-mode” of mind
	**discussing the cognitive model by Salkovskis**, **emphasizing the impact of the subjective appraisal of thoughts**
	diary of positive and negative events
	**discussing neurobiological correlates of OCD to encourage disidentification with obsessive thoughts and impulses**
Freeing oneself from dysfunctional habits and being kind with oneself	**identifying individual OCD triggers**
	**encouraging participants to perform the three**-**minute**-**breathing**-**space whenever they notice obsessive thoughts and impulses**
	sharing poems, short stories and metaphors in order to invite participants to experience thoughts as passing events of the mind rather than reflections of reality
Relapse prevention	establishing individual schemes for dealing with difficult situations
	setting objectives for goal-oriented behavior
	integrating pleasant activities into everyday lives

OCD-specific contents are highlighted in bold type.

MBCT trainers (NR, AKK) were experienced cognitive behavioral therapists with several years’ expertise in mindfulness practice. They had been trained by one of the founders of MBCT, Mark Williams. Sessions were videotaped and supervision was provided by TH.

### Data collection and analysis

Interviews and data analysis were conducted by a staff member experienced in the area of OCD who was familiar with the MBCT manual. Based on an extensive literature search, a semi-structured interview schedule was developed by the workgroup, covering the main and secondary research questions. The interview schedule consisted of the following questions:

1) When you think about the past eight weeks and the mindfulness program, what comes to your mind?

2) Did you notice any kind of changes over the past eight weeks?

3) How did you experience mindfulness?

4) Do you think that mindfulness was helpful regarding your OCD?

5) Did you experience any difficulties?

6) If you could change anything about the program, what would you change?

Due to the fact that hardly any literature is available on subjective experiences of patients with OCD undergoing mindfulness training, the interview schedule was used only to roughly structure conversations, allowing to digress into other topics brought up by the participants (in accord with Smith, [24]). Each interview lasted approximately 30 minutes and was videotaped, or, in two cases, audiotaped, as participants refused being videotaped.

For data analysis, an inductive data-driven approach was chosen. It allows the extraction of core themes, as recommended for example by Hsieh and Shannon [25] for cases “when existing theory or research literature on a phenomenon is limited”, and in line with previous studies on similar research questions (e.g. [6,8]). Precisely, qualitative content analysis following Mayring [26] was selected because it is a widely and successfully used method (compare e.g. [27,28]) and because it was considered a rather economic technique, compared for example to grounded theory. Based on principles and techniques suggested by Mayring [26], a multistage analytic strategy was developed: First, video- and audiotapes were transcribed verbatim. In the course of reading and re-reading transcripts, text passages that appeared to be relevant with regard to the research questions were extracted and coded, i.e. labeled as a term preferably close to the text passage itself. Further relevant text passages were either subsumed under an existing term or, whenever they did not fit into an existing category, assigned a new term. In this vein, the first seven transcripts were coded. The emerging system of categories was then discussed in an audit within the working group and partially revised. Subsequently, the remaining five interviews were coded. The resulting system of numerous categories, which still closely matched the verbatim data, was again revised following discussions within the workgroup. Similar categories were grouped to extract a smaller number of abstract, overarching themes. Qualitative content analysis resulted in a system of five categories and several subcategories which were assumed to sufficiently represent the data material.

In order to bolster the qualitative reports with data from another source, we administered a self-developed questionnaire assessing the frequency of mindfulness practice and the participants’ satisfaction with the individual mindfulness exercises (body scan, sitting meditation, yoga, three-minute breathing-space). Every treatment completer filled in the questionnaire immediately after the last MBCT session. The patients were asked how many MBCT sessions they had attended, how often they had practiced mindfulness outside the sessions, and were requested to rate every mindfulness exercise on a five-point Likert scale ranging from “very helpful” to “not at all helpful”.

## Results

### Qualitative reports

All 12 treatment completers appreciated the program as a whole, were glad they had participated and stated that they had in one way or another experienced it as an enrichment to their lives. Five overarching categories, three of them divided into two subcategories each, emerged from the data analysis. Due to the semi-structured style of the interviews, not every participant commented on every theme. Table 2 summarizes the categories and subcategories (subcategories in italics) and shows the number of patients who commented on the respective themes.

**Table 2:** System of five overarching themes that emerged from qualitative content analysis

**Theme**	**N** =	**summary**
Being in a group		
*Other participants*	10	Prior to the course, six participants had been doubtful regarding the group format. Over the course of the program, however, a sincere and trusting atmosphere developed and participants experienced the exchange with others as helpful. Connecting with the other participants affected by OCD served as an important self-validating experience.
*MBCT trainers*	11	Trainers were rated as authentic, committed and professional. Participants could especially benefit from the trainers’ own personal experiences with mindfulness that were repeatedly reported during the sessions.
Mindfulness Exercises		
*Evaluation*…	8	Three participants found the three-minute-breathing-space especially helpful. Patients liked that mindfulness exercises were not performed with a defined goal in mind, and that it was said that wandering of the mind is a natural process. One participant distinguished outer and inner mindfulness, and found exercises referring to outer mindfulness especially helpful.
…*practical implementation*	10	The three-minute-breathing space was perceived as particularly feasible, whereas it was difficult to implement the longer exercises into everyday life. The bodyscan and the yoga exercises were often conducted before going to bed, in order to come to rest. Waiting periods were experienced as convenient for practicing mindfulness. Several participants have slightly modified the exercises to fit their own needs.
Effects		
*benefits*	11	Nine participants described new strategies they had learned to deal with their OCD, most of them resulting in an increased ability to refrain from compulsive rituals. Reported benefits furthermore included an increased willingness to experience unpleasant emotions and insights into the nature of the mind, living more actively in the present moment, a calmer attitude, improved mood and improved sleep.
*undesired outcomes*	6	Four participants stated that regarding their OCD, the course had not been helpful. One patient repeatedly experienced a highly unpleasant state performing the body scan. Another participant suffered from the experience that compared to the other patients in the program, he was far more severely affected with OCD.
Struggle	11	Three participants described that OCD symptoms repeatedly conflicted with their mindfulness practice, making it difficult to get themselves into the exercises. Whereas most participants found it rather easy to do a three-minute-breathing-space at a previously defined time, a common difficulty was to remember the exercise early enough in difficult situations related to OCD. Further difficulties included dealing with one’s expectations regarding mindfulness as a fast “cure”, as opposed to being a fairly long-term, fundamental approach.
Modifications	9	Participants’ ideas to modify MBCT mainly concerned the length of the program: Several attendees suggested to either extend the length of the individual session beyond 120 minutes, or to add further sessions. One participant proposed to schedule longer meditation exercises towards the middle of the sessions, as he found it difficult to engage in meditation directly after arrival.

N = number of patients who commented on the respective theme. Subthemes are written in italics.

Two of the five categories, namely “benefits” and “struggle”, will be described in more detail and will be illustrated using quotations from the interviews. These two categories were chosen because patients’ experiences of benefits and struggles in the context of MBCT appear to be of particular clinical interest. Patients’ reports about the other participants and the MBCT trainers, in contrast, reflect unique characteristics of our study setting. Please note that quotations have been translated from German to English. The statements have been minimally edited to retain the raw, spontaneous nature of the original but also make for smoother reading.

#### Benefits

Nine participants stated that during MBCT they had become acquainted with a new way of dealing with their OCD. One patient described how he managed to reduce his washing compulsions with the help of a mindfulness technique:

“When this urge comes, like let’s say, I want to step out right now and wash my hands, that I then first pause for a second and remind myself to also be mindful with myself, that I then say – in the past I would have usually just bolted off right away – and now when I first pause for a moment, this has been an important word for me during these eight weeks, an important insight, then I can say, yes, just kinda like it says in your book or in these scripts, then I can say I will finish this here first and after half‘n hour this impulse to leave maybe doesn’t appear at all anymore, and if it does I’ve still accomplished something, at least that‘s how I feel about it.”

Another participant found the three-minute breathing space especially helpful in dealing with his compulsive rituals:

“Well, like, you know, in some stressful situations, or when I, you know, thought that ok, now I should actually act all compulsive, well, then I just have, for example, well, that I’ve just frequently applied this three-minute breathing space. Well, also to find out, what do I actually really want? And, do I have to do it now, or… Then, sometimes I nonetheless acted out the compulsion, but not as long as I used to, well, like before I took the program. Looking at it this way, it really did a lot for me. Yes. And it, like, also distracts you. Because then my thoughts aren’t on the compulsion anymore, but rather first on like: Where am I right now, what am I doing, and why. That was actually good, yeah.”

A participant who predominantly suffered from obsessions with aggressive and sexual content also successfully used mindfulness techniques:

“Well, and it is like simply ‘n alternative to suppressing and to “obsessively”, yeah “obsessively” thinking of positive things. Well, which just uses up an insane amount of energy, too. And this staying mindful, and then just saying what is actually going on right now, just seems to make a lot more sense to me. Well, I notice that this is definitely a very good way. Maybe even the only one that exists that could really help. But it requires a great deal of practice, and you have to, how should I say, constantly make yourself aware of it and also tell yourself all the time ok now stay here, don’t chase your thoughts, just let them pass by. This works. It works, but it’s strenuous.”

For this patient, information and discussion about the nature of the mind and thoughts were of particular interest. Realizing that having sudden unpleasant, intrusive thoughts is a common phenomenon known not just to patients with OCD but to most people, meant a great relief to him and made it easier for him to observe his obsessions without judging himself for their occurrence. Accordingly, he tried to mindfully watch obsessive thoughts come and go, to make them out as obsessions and then to bring his attention back to the present moment. A woman affected by dirt- and blood- related obsessions as well as washing and cleaning compulsions explained that being mindful leads to a decreased occurrence of obsessions:

“Of course, because, when I go shopping, just as an example, I go shopping. And then, everything else is not important in that moment. I just go shopping and focus on that. Well that’s how I feel about it now. If I really put myself into that, then there is not much space for other things.”

Furthermore, the patients reported an increased willingness to tolerate even unpleasant emotions when performing mindfulness meditation. Three participants reported that feelings such as sadness and pain occurred during the body scan. All of them stated that they had been able to let these feelings surface without trying to change them or get rid of them. Four participants reported that MBCT had taught them to live more actively in the present moment. Consequently, they perceived a reduced tendency to ruminate and worry as well as an increased ability to engage in pleasant activities and to value these experiences. One patient noted that whilst MBCT had not helped her to reduce her obsessions and compulsions, she experienced herself as reacting calmer even in difficult situations, more capable to accept her OCD as a part of her life and as being able to enjoy her life despite her struggle with OCD. Last but not least, some participants specifically used mindfulness exercises before going to bed and reported a positive impact on their sleep.

#### Struggle

Three participants reported that OCD symptoms considerably interfered with their mindfulness practice during and beyond the sessions. One patient stated that at the beginning of the program it had been extremely challenging just to sit in a room together with the other participants because, as she stated, “every breath of air was OCD”. By the end of the course, however, she noticed that considerably fewer counting compulsions occurred during the sessions. She reported that during the eight weeks it had become easier for her to restrain herself from engaging in rituals, though she was still afflicted with several obsessive concerns. Another participant suffered from extensive mental compulsive rituals. More precisely, he felt the urge to mentally repeat everything that he or others had said. Consequently, he found it extremely difficult to stop himself from repeating the trainers’ instructions over and over during mindfulness exercises. Three patients found it especially challenging to use mindfulness techniques such as the three-minute breathing space as a skill in difficult situations related to OCD. One participant explained that he experienced his compulsions as a highly automatized behavior – he repeatedly caught himself in the act, becoming aware then that he had not been able to mindfully notice an obsessive urge before engaging in a compulsive ritual. Three more participants found the mismatch between their wish to get rid of their OCD as fast as possible and mindfulness being a long-term process troubling. Adding to this is that mindfulness initially does not appear goal-oriented. One participant phrased it this way:

“On a mental level, I sometimes have problems that I start thinking, with the body scan, and so on: And that’s it now? One somehow always expects something to happen immediately. But here, that’s just it for the moment, and let it sink. Yes. That you shouldn’t hang your hopes so - basically not to have any expectations at all, I think this seems to be the important point. Rather, it just happens automatically. And you have to be patient there, with yourself.”

### Ratings

Of a total of twelve treatment completers, four attended all eight MBCT sessions, five missed one session and three missed two. Two participants indicated that they had practiced mindfulness outside the sessions six to seven times per week. Five participants reported that they had practiced four to five times and the five remaining participants had practiced two to three times.

Concerning the ratings of satisfaction, the three-minute-breathing space received the most favorable ratings: nine out of twelve participants reported that this exercise was “very helpful” for them and one participant rated it as “quite helpful”. Eight patients found the body scan very or quite helpful, seven said this about the sitting meditation and four about the yoga exercises.

## Discussion

The results of the present pilot study provide preliminary evidence that patients with OCD find aspects of MBCT acceptable and beneficial.

Changes within eight weeks included a perceived decline of obsessive compulsive symptoms, living more actively in the present moment, an increased ability to allow unpleasant emotions, a calmer attitude in everyday life and towards OCD as well as improved mood and sleep. Four out of twelve participants reported that they did not observe any improvement of OCD symptoms after MBCT.

Concerning helpful and problematic elements of MBCT, the patients’ reports indicate that the three-minute breathing space might be a particularly helpful element of MBCT in OCD, at least short-term. Our data do not reveal harmful secondary effects of MBCT in OCD. One participant repeatedly became aware of unpleasant emotional and physical states during the body scan. However, as he stated, this was not a purely undesired outcome, as he tried to embrace these states as opportunities to relate differently to unpleasant feelings and sensations. He reported that the body scan sometimes even brought to light inner struggles that had been rather unconscious. This helped him clarify problems he was currently dealing with. Problematic aspects included that the patients’ struggle with OCD symptoms interfered with their mindfulness practice, difficulties applying mindfulness techniques as a skill in OCD-related situations and the perceived discrepancy between their desire for change and the accepting attitude inherent in the mindfulness concept.

We showed that the MBCT manual as adapted for OCD by our workgroup can be applied to patients with acute OCD. MBCT was originally designed as a relapse prevention for patients in remission from major depressive disorder, that is, for participants who are not presently symptomatic. Our findings suggest that patients with acute OCD, despite various reported difficulties, can also benefit from the approach. Several participants suggested continuing the program after the eight sessions, and pointed out possible difficulties in implementing mindfulness in everyday life without guidance and feedback. One way of implementing these recommendations is to provide so called “booster sessions” or “retreats” several months after the end of the actual program, as planned within the scope of our project and also reported for example by Chadwick et al. [8].

The present analysis allows preliminary conclusions about components of MBCT that might be useful and/or difficult for patients with OCD. Deeper knowledge about particular treatment components might in turn allow revisions of the treatment manual, e.g. focussing on therapeutic elements that are especially likely to produce desired changes. The qualitative reports as well as the ratings of satisfaction indicated that our participants especially valued the three-minute breathing space. The breathing space is a “mini-meditation” which can help those practicing it to reconnect themselves with the present moment. Several patients reported that with the help of the breathing space, they were able to “pause” for a moment when they felt a compulsive urge. This resulted in a heightened ability to regulate compulsive behavior, whereas prior to MBCT, compulsions had been perceived as rather uncontrollable. This may be due to an improved self-regulation of attention, resulting in a) being more aware of internal events, for example recognizing compulsive urges at an earlier stage and b) being more able to purposefully redirect attention, e.g. away from intrusive thoughts, focussing on one’s breathing and thus interrupting dysfunctional stimulus–response-chains.

Another component of MBCT that our participants perceived as helpful was the acceptance of private experiences and self-acceptance. The patients reported that after MBCT, they were able to handle their emotions more flexibly and with an increased willingness to experience unpleasant states. This seems especially relevant for patients with OCD since obsessive-compulsive symptoms frequently serve as processes that regulate unpleasant emotions [29]. The accepting attitude did not exclusively relate to emotions: Some participants also reported that they had learned to relate to their obsessions in a different way, e.g. recognizing them as fluctuating events of their mind and letting them pass by without devaluing themselves. Whereas MBCT and the meta-cognitive therapy (MCT), which has also recently been applied to patients with OCD [30], share the objective of changing the attitude towards private experiences rather than their content, acceptance, self-acceptance and the non-judging attitude are more specific for mindfulness and MBCT. In our sample, the acceptance-focused elements of MBCT were perceived as beneficial. This finding appears plausible from a theoretical point of view, as OC symptoms have been linked to pathological guilt [31] and shame-proneness [32]. Acceptance-based interventions like MBCT might be especially useful to directly target these factors of OCD.

Thus, we recommend that future MBCT protocols for patients with OCD highlight the three-minute breathing space, self-acceptance and the acceptance of private events.

Comparing our results to findings on subjective experiences of patients with mood disorders who underwent MBCT, we found several parallels. Finucane and Mercer [6] extracted “being in a group”, “the course exercises” as well as “benefits and ongoing practice” as important over-arching themes from their data. As in our study, they point out that the program was felt to be too short. A “dialectic tension” between the wish for change on the one hand and an accepting attitude on the other hand has previously been reported by Allen et al. [5], who interviewed patients with recurrent depression one year after MBCT. Chadwick et al. [8] found that patients with bipolar disorder undergoing MBCT reported earlier and clearer awareness of mood shifts, allowing them to prevent escalation into depression or mania. Similarly, our study participants were able to “pause” when they recognized a compulsive urge, instead of uncontrollably slipping into automated behavior. Interestingly, other researchers chose longer intervals between the end of the program and the interviews, allowing for an exploration of medium-and long-term effects of mindfulness training. Allen et al. [5], for example, found changes in interpersonal relationships attributed to MBCT. Finucane and Mercer [6] report that the program helped two of their participants to return to work. It cannot be ruled out that mindfulness training elicits these changes only in patients with mood disorders and not in OCD patients; however, it seems reasonable that changes in social relationships and work life are fairly long-term effects that were not addressed by our short-term approach. Findings by Williams et al. [9] who interviewed patients with hypochodriasis three months after MBCT also showed considerable parallels to our findings. They report that patients benefited from mindfulness training through understanding and breaking their health anxiety circles, accompanied by a decrease in automatically reacting to bodily sensations and anxious thoughts. This closely resembles reports from OCD patients in our study who were better able to interrupt dysfunctional stimulus–response-chains. Williams et al. [9] concluded that benefits of MBCT extended beyond the reduction of health anxiety symptoms to more widespread effects like a more accepting attitude, an increased ability to relax, improved mood and sleep. Our results show the same effects.

Comparing our results to a qualitative study on patients with OCD who received CBT [33], we observed that the patients apparently perceive both CBT and MBCT as helpful in dealing with their OCD. Similar to our sample, the twelve patients in the study by Bevan et al. [33] reported that their overall experience with the treatment was very positive. The authors focused on the comparison of an intense form of CBT to a weekly format and did not explicitly focus on treatment components that were perceived as helpful or difficult. Therefore, only limited comparisons to our results are possible.

Our study has several strengths, but also clear limitations. To our best knowledge, the present open pilot study is the first research project that applies MBCT to OCD patients. Against this background and considering the explorative character of our research questions, a qualitative methodology appeared appropriate. To support these findings with more objective data, clinician ratings of OCD severity, depressive symptoms and global level of functioning were recorded at baseline, post and 6 months follow-up. These results will be reported elsewhere. In our analysis, we adhered to quality criteria defined by Mayring [26]: we chose a semi-structured interview style and an inductive analytic approach in order to have an open mind to any emerging aspects not previously considered. Data analysis followed a systematic rule-governed procedure involving a team of researchers, thus ensuring a certain degree of inter-subjectivity of our findings. However, qualitative methodology remains a rather subjective approach, reflecting the experiences of the studied subjects only, and is prone to researcher bias. Interpretability and generalizability of the reported findings are further limited by the lack of a control group, the small sample size and the unbalanced gender ratio. Furthermore, patients’ reports may have been inflated by a tendency to answer in a socially desired manner. The fact that interviews were conducted directly after the course limits our statements to a short-term perspective, lacking information about long-lasting effects of mindfulness training in OCD. However, scheduling interviews directly after the course probably precluded memory bias.

## Conclusion

In summary, we showed that MBCT can be applied to OCD patients experienced with exposure and response prevention, that patients report various benefits and experience no major harmful outcomes. However, our study provides only preliminary results, leaving open a number of questions to be considered in the future. First, randomized controlled trials with larger sample sizes are needed to evaluate the efficacy of MBCT in OCD and specify the effects of the program in comparison to active control groups. If the results of such a trial should be in favor of MBCT, it needs to be further elucidated how MBCT fits into the current health care system. In the present report MBCT was applied to patients who had already received CBT with ERP. Consequently, and due to the pilot character of our study, no reliable statements can be made as to whether MBCT should best be delivered as a complementary treatment to patients who do not or only partially respond to ERP, as an add-on for patients who undergo ERP at the same time, as a preparation to ERP for patients who are reluctant to engage in ERP, or as a stand-alone treatment. Second, the outcome variables that appropriately and fully capture the changes elicited by MBCT need to be determined. Our findings suggest that mindfulness training may not only help alleviate OCD symptoms, but may lead to benefits beyond symptom reduction. This implicates that further research on mindfulness treatment in OCD should not be limited to measures of symptom severity. It should additionally, in a more holistic approach, address further outcome measures such as the quality of life as well as possible mediating variables such as an accepting attitude, the self-regulation of attention, and meta-cognitive beliefs.

## Competing interests

CN has received speaker honoraria from Servier and has served as a scientific advisor for Novartis. EH, NR, TH, NH, NS, AKK and UV report no financial or non-financial competing interests.

## Authors’ contributions

EH acquired and analyzed the data and drafted the manuscript. AKK planned and conceptualized the study, designed the treatment manual and was involved in the interpretation of the results and in preparing the manuscript. NR and TH co-conceptualized the study, co-designed the treatment manual, were involved in the interpretation of the results and critically revised the manuscript. CN, NS, NH and UV were involved in the study conceptualization, interpretation of the results, discussion and revision of the manuscript. All authors read and approved the final manuscript.

## Pre-publication history

The pre-publication history for this paper can be accessed here:

http://www.biomedcentral.com/1471-244X/12/185/prepub
